# Effect of Encapsulation on the Viability of *Lacticaseibacillus rhamnosus* and *Lacticaseibacillus paracasei* During In Vitro Gastrointestinal Digestion and Storage Conditions

**DOI:** 10.1002/fsn3.70614

**Published:** 2025-07-16

**Authors:** Elif Tülek, Büşra Karkar, Saliha Şahin, Lütfiye Yılmaz‐Ersan, Ekin Sucu

**Affiliations:** ^1^ Department of Chemistry, Faculty of Science and Arts University of Bursa Uludağ Bursa Türkiye; ^2^ Department of Food Engineering, Faculty of Agriculture University of Bursa Uludağ Bursa Türkiye; ^3^ Department of Animal Science, Faculty of Agriculture University of Bursa Uludağ Bursa Türkiye

**Keywords:** carob flour, encapsulation, functional food, *Lacticaseibacillus paracasei*, *Lacticaseibacillus rhamnosus*, optimization

## Abstract

Probiotic microorganisms are vital for gut health, but their viability is compromised by environmental stressors such as gastric acidity, bile salts, temperature, and oxygen. Encapsulation is a promising strategy to enhance probiotic stability and targeted release. In this study, *Lacticaseibacillus rhamnosus* and *Lacticaseibacillus paracasei* were encapsulated using an emulsion method with carob flour, a natural material with prebiotic potential. Encapsulation conditions were optimized using the Box–Behnken Design, considering mixing temperature, mixing time, and carob flour amount. The encapsulation efficiencies were 79.51% ± 0.36% for *L. rhamnosus* and 74.98% ± 0.20% for *L. paracasei* . Encapsulation induced significant compositional and structural changes, as confirmed by physicochemical, FTIR, and SEM analysis. Encapsulated probiotics exhibited higher viability than free bacteria during in vitro gastrointestinal digestion and under refrigerated and frozen storage. Encapsulation with carob flour is a promising approach to enhance probiotic stability and bioavailability in functional food applications.

## Graphical abstract

This study explores the encapsulation of *L. rhamnosus* and *L. paracasei* using carob flour via an emulsion method. The encapsulated probiotics demonstrated significantly improved survival under gastrointestinal and storage stress conditions. The optimized process was evaluated through physicochemical, FTIR, and SEM analysis, revealing successful structural integration and stability enhancement.

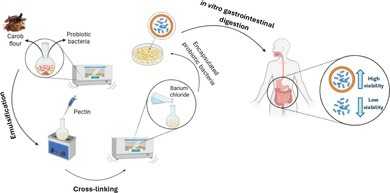

## 1 Introduction

In recent years, as consumers have become more aware of their dietary preferences, health maintenance through nutrition has become increasingly popular. This trend has accelerated the expansion of the food sector, particularly with the development of probiotic‐enriched functional products designed to promote gastrointestinal health. Probiotic microorganisms are predominantly incorporated into fermented dairy products, such as yogurt, cheese, milk‐based formulations, and chocolate and infant formulas (Stanton et al. 2005; Uymaz 2010; Kaur et al. 2022). The efficacy of probiotic‐containing foods depends on maintaining a minimum viable microorganism count, as probiotics must remain in sufficient numbers to exert their beneficial effects. Generally, it is recommended that the bacterial viability in probiotic products be at a level of 10^6^–10^7^ cfu/mL (g), and 10^6^–10^9^ cfu/mL (g) probiotics should be taken with daily consumption (Gismondo et al. 1999; Bilginer and Çetin 2019). The principal bacterial genera used in probiotic formulations belong to *Bifidobacterium* *Lacticaseibacillus* and *Lactobacillus*, which are resident species of the human gut microbiota (Uymaz 2010; Latif et al. 2023). The viability and functionality of probiotics are profoundly influenced by various environmental stressors, including acidic conditions, oxidative stress, storage temperature, microbial competition, osmotic stress, and variations in moisture content (Stanton et al. 2005; Luo et al. 2022). Encapsulation has emerged as a biotechnological approach involving the coating of probiotic microorganisms with a protein, carbohydrate, or lipid based protective material to protect them from various stress factors (Gökmen et al. 2012; Serna‐Cock and Vallejo‐Castillo 2013; Roshanzamir et al. 2017). This method enhances the stability of probiotics during storage and processing, facilitating their targeted release within the gastrointestinal tract (Geniş and Tuncer 2019; Gökmen et al. 2012). Encapsulation methods employed in the food industry include extrusion, emulsion, spray drying, and phase separation, all of which have demonstrated efficacy in preserving probiotic viability (Ananta et al. 2004; Uran et al. 2017; Latif et al. 2023). The emulsion method involves suspending probiotic bacteria within an emulsion matrix, followed by polymer‐based encapsulation utilizing carrageenan, carob bean gum, alginate, chitosan, or gelatin. This approach is widely used to encapsulate probiotics within water‐in‐oil (W/O) or oil‐in‐water (O/W) emulsions. Double‐emulsion methods, such as W/O/W or O/W/O, modify the basic method. These emulsions commonly use vegetable oils, such as canola, sunflower, or corn oil. To further enhance capsule stability, a cross‐linking agent is incorporated, which facilitates the formation of a robust protective barrier that shields the probiotics from adverse environmental conditions (Kailasapathy 2002; Chávarri et al. 2012; Gökmen et al. 2012; Choińska‐Pulit et al. 2015; Eghbal et al. 2022). In addition to these technological advancements, probiotics provide health benefits by interacting with prebiotics, which are fermentable dietary fibers that promote the selective growth of beneficial gut bacteria (Bilginer and Çetin 2019; Salçın and Ercoşkun 2021). The beneficial effects of prebiotics are mostly pronounced when used at the correct dose, making their incorporation into food products essential. Fructooligosaccharides, galactooligosaccharides, and trans‐galactooligosaccharides are among the most utilized prebiotics. Numerous studies have underscored the efficacy of encapsulation in polysaccharide matrices as a highly effective strategy for ensuring probiotic viability during processing, storage, and gastrointestinal transit (Davani‐Davari et al. 2019; Ferreira et al. 2023; Ali et al. 2023).

Carob ( *Ceratonia siliqua* L.) belongs to the *Leguminosae* (*Fabaceae*) family. It is a plant that has been cultivated since ancient times in most countries of the Mediterranean and Aegean regions due to its agronomic resilience and nutritional richness (Petkova et al. 2017; Pazır and Alper 2018; Zhu et al. 2019). The carob fruit is brown and can grow up to 25 cm under certain conditions (Kumazawa et al. 2002). The fruit comprises 90% edible parts and 10% seeds (Pazır and Alper 2016; Zhu et al. 2019). Carob flour is obtained from the pods and seeds of the carob fruit (Petkova et al. 2017). The chemical composition of carob fruit varies depending on its variety, origin, and harvest time. It is rich in sugar, dietary fiber, minerals, and phenolic compounds. Carob is a low‐glycemic index food because of its high insoluble fiber content. It is associated with cardiovascular health benefits, especially in lowering cholesterol levels. High‐fiber diets promote colon health by supporting beneficial gut microbiota and regular bowel movements (Pazır and Alper 2016, 2018). Due to its functional phytochemical components, aromatic characteristics, and nutritional benefits, carob has significant potential for use in various industries, including food, textiles, cosmetics, and pharmaceuticals (Kumazawa et al. 2002; Petkova et al. 2017; Brassesco et al. 2021).

This study aimed to determine whether probiotic bacteria can maintain/increase their viability during storage and gastrointestinal digestion after consumption. To achieve this, *Lacticaseibacillus rhamnosus* and *Lacticaseibacillus paracasei* probiotic bacteria were encapsulated using carob ( *Ceratonia siliqua* L.) flour, which is known for its high nutritional value and potential prebiotic properties. The encapsulation process was optimized using the Box–Behnken Design‐Response Surface Methodology (BBD‐RSM) to identify the best conditions for encapsulation. The morphological structure and physicochemical properties of the probiotic bacterial capsules produced under these optimal conditions were examined. Additionally, the study evaluated the ability of these probiotic capsules to maintain their viability during storage and simulated gastrointestinal digestion. The results were compared to the viability of free probiotic bacteria.

## 2 Materials and Methods

### 2.1 Sterilization of Carob Flour

Carob flour (CF) was commercially purchased from a natural food product manufacturer in Antalya, Turkey. Sterilization of the CF, which serves as the coating material, is crucial before encapsulation to prevent contamination. However, the sterilization time must be meticulously controlled to preserve the material's structural integrity and chemical composition. The sterilization time was set to 1 min at 121°C, as determined to be optimum in the literature (Karkar et al. 2024).

### 2.2 Activation of Probiotic Bacterial Strains

*Lacticaseibacillus rhamnosus* (LGG) and *Lacticaseibacillus paracasei* (L. CASEI 431) (Chr. Hansen A/S, Hørsholm, Denmark) were selected as probiotic bacteria. De Man Rogosa and Sharpe (MRS) Broth, MRS Agar, and all materials and solutions were sterilized at 121°C for 15 min. The bacterial strains were incubated three times in MRS Broth liquid medium to activate them (for 24 h at 37°C) under anaerobic conditions. After incubation, the bacterial cultures were stored at −24°C.

### 2.3 Encapsulation of Probiotic Bacteria With CF

**FIGURE 1:**
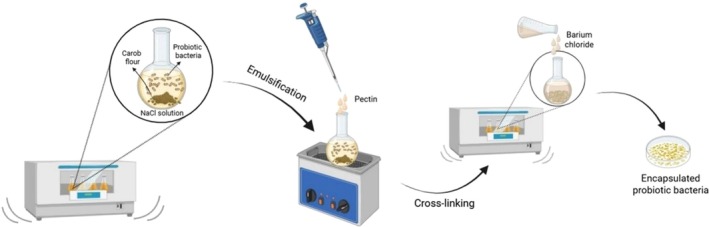
Schematic representation of the emulsion method.

The encapsulation of *L. rhamnosus* (LR) and *L. paracasei* (LPC) probiotic bacteria with CF was carried out using the emulsion method, incorporating modifications based on the methods described in the literature (Homayouni et al. 2007; Karkar et al. 2024). The schematic diagram illustrating the emulsion method used is displayed in Figure 1. Prior to encapsulation, the probiotic bacteria were activated by transferring 1 mL from stock bacterial cultures into 25 mL of MRS broth and incubating at 37°C for 24 h (Karkar et al. 2024). Once activated, the probiotic cultures were added to sterilized CF at a concentration that ensured viability of at least 10^6^–10^7^ cfu/mL (Gismondo et al. 1999). Following this, 50 mL of a 0.5% (w/v) NaCl solution was introduced, and the mixture was stirred on a temperature‐controlled shaker at 100 rpm. Next, 2.5 mL of a 1% (w/v) pectin solution was added, and the mixture was held in an ultrasonic bath at 25°C for 20 min. After this period, the mixture was centrifuged at 3000 rpm for 15 min to separate the supernatant. Next, 10 mL of a 0.5% (w/v) BaCl_2_ solution was added to the medium to perform cross‐linking, and the mixture was stirred for 30 min. A second centrifugation was then performed at 3000 rpm for 15 min, after which the supernatant was carefully decanted. Finally, the capsules were lyophilized at −86°C under a pressure of 0.1 mbar.

### 2.4 Microbiological Analysis of Probiotic Bacteria

The microbiological analysis of free and encapsulated LR and LPC was performed using the pour plate method (Chandramouli et al. 2004). The capsules were opened to release the bacteria and assess the viability of probiotic bacteria within them. A pH 7.0 ± 0.2 phosphate buffer (0.1 M K_2_HPO_4_/KH_2_PO_4_) was prepared for this purpose. Capsules were kept in the buffer for 30 min and vortexed every 5 min. Serial dilutions (10^−1^ to 10^−8^) were prepared using physiological saline solution (0.85% NaCl, w/v). Aliquots of 1 mL from each dilution were placed into sterile petri dishes. The MRS (De Man, Rogosa, and Sharpe agar, Merck, Germany) media were used to determine Lactobacillaceae counts. MRS agar plates were incubated at 37°C under anaerobic conditions (Thermo Scientific Oxoid AnaeroGen, UK) for 72 h. The viability of encapsulated probiotic bacteria was expressed as a log cfu/g, whereas the viability of free probiotic bacteria was expressed as a log cfu/mL (Tharmaraj and Shah 2003; Karkar et al. 2024).

### 2.5 Optimization of Encapsulation Conditions

Achieving maximum efficiency in the encapsulation of probiotic bacteria with CF, the encapsulation conditions were optimized using the Box–Behnken Design (BBD) from chemometric methods (Yağmur and Şahin 2020). The critical factors chosen were mixing temperature (°C), mixing time (min), and CF amount (g). The factors and coded level values used for BBD are provided in Table 1. The number of experiments (N) for a three‐level, three‐factor design with five repetitions at the center point was calculated using Equation (1).

(1) $$N=2k\left(k-1\right)+C_{k}$$

where *k* represents the number of factors, and *C*_*k*_ represents the center points.

**TABLE 1:** Factors and coded level values for Box–Behnken Design‐response surface methodology.

Factor	Coded level values		
	−1	0	1
(*x*_*1*_) Mixing temperature (°C)	25	30	35
(*x*_*2*_) Mixing time (min)	15	45	75
(*x*_*3*_) Carob flour amount (g)	1	2	3

In order to optimize these three independent factors, response surface methodology (RSM) combined with BBD was applied using 17 experiments for both probiotic bacteria, as detailed in Table 2. The encapsulation efficiency for each experiment was calculated using Equation (2). The response values were analyzed using analysis of variance (ANOVA) with Design‐Expert 7.0.0 (Stat‐Ease Inc. USA).

(2) $$\text{Encapsulation efficiency}\;\left(\%\right)=\frac{\log N_{\text{encapsulated}}}{\log N_{\text{free}}}\times 100$$

where *N*_encapsulated_ represents the encapsulated probiotic bacteria count (cfu/g), and *N*_free_ represents the free probiotic bacteria count (cfu/mL).

**TABLE 2:** Box–behnken design table.

Run order	Factor		
	*x*_*1*_	*x*_*2*_	*x*_*3*_
	Mixing temperature (°C)	Mixing time (min)	Carob flour amount (g)
1	25	15	2
2	35	15	2
3	25	75	2
4	35	75	2
5	25	45	1
6	35	45	1
7	25	45	3
8	35	45	3
9	30	15	1
10	30	75	1
11	30	15	3
12	30	75	3
13	30	45	2
14	30	45	2
15	30	45	2
16	30	45	2
17	30	45	2

In the experimental study, ANOVA analysis was conducted, and estimated values were calculated using a second‐degree equation (Equation 3).

(3) $$y=b_{0}+\sum_{i=1}^{3}b_{i}x_{i}+\sum_{i=1}^{3}b_{\mathrm{ii}}x_{i}^{2}+\sum_{i=1}^{2}\sum_{j=i+1}^{3}b_{\mathrm{ij}}x_{i}x_{j}$$

where *y* represents the response variable, *b*_0_ is the constant term, *bᵢ* is the linear interaction coefficient, *bᵢᵢ* is the quadratic interaction coefficient, *bᵢⱼ* is the interaction term coefficient, and *xᵢ* and *xⱼ* are the independent factors.

The optimum values for each factor were determined, and under optimum conditions, encapsulation was performed to calculate the encapsulation efficiency. The experimentally determined encapsulation efficiency was then compared with the predicted efficiency values derived from the model. The RSM was utilized to evaluate the effects of binary interactions among factors on encapsulation efficiency through response surface plots.

### 2.6 Physicochemical Analysis of CF‐Probiotic Bacteria Capsules

The physicochemical properties of CF and CF‐probiotic bacteria capsules, including total sugar (AOAC 968.28 and TS 1466 modified), total fat (TS EN ISO 659), total protein (AOAC 990.03), dietary fiber (AOAC 991.43), dry matter and moisture (TS 1129 ISO 1026), titratable acidity (TS 1125 ISO 750), and ash (TS EN ISO 2171) were analyzed with standard methods.

### 2.7 Characterization of CF‐Probiotic Bacteria Capsules

The morphological analysis of CF, sterilized CF, and CF‐probiotic bacteria capsules was examined using Scanning Electron Microscopy (SEM) (Carl Zeiss Evo 40) at an accelerating voltage of 10–20 kV. Structural characterization of pectin, CF, sterilized CF, and CF‐probiotic bacteria capsules was analyzed using a Fourier Transform Infrared (FTIR) spectrometer (PerkinElmer, Spectrum 100).

### 2.8 Viability of Probiotic Bacteria During In Vitro Gastrointestinal Digestion

The viability of free and encapsulated LR and LPC in simulated gastric fluid (SGF) and simulated intestinal fluid (SIF) was evaluated using methods modified from those in the literature (Tipigil 2015; Karkar et al. 2024). SGF was prepared by dissolving 1 g of NaCl and 1.6 g of pepsin in sterilized distilled water, with the final volume adjusted to 500 mL. The pH was adjusted to 2.0 ± 0.2 using 0.2 N HCl. SIF was prepared by dissolving 3.4 g of KH_2_PO_4_, 38.5 mL of 0.2 N NaOH solution, 0.625 g of pancreatin, and 1.5 g of bile salt in sterilized distilled water, with the final volume adjusted to 500 mL. The pH was adjusted to 7.0 ± 0.2 using 0.2 N NaOH and 0.2 N HCl. The prepared SGF and SIF media were passed through a 0.45 μm single‐use sterile PVDF filter and stirred at 37°C for 1 h. Then, encapsulated probiotic bacteria (1 g) and activated free probiotic bacteria (1 mL) were introduced into 19 mL of SGF and SIF media. The mixture was incubated at 37°C with continuous stirring at 100 rpm for 30, 60, 90, and 120 min. After the gastric digestion phase was completed, the pH was adjusted to 7.0 using 0.2 N NaOH, and 20 mL of SIF was added. The mixture was subsequently incubated at 37°C under shaking conditions for 30, 60, 90, and 120 min to simulate intestinal transit. The impact of in vitro gastrointestinal digestion on the viability of free (log cfu/mL) and encapsulated (log cfu/g) probiotic bacteria was determined using Equation (4).

(4) $$\text{Viability}\;\left(\%\right)=\frac{N_{\text{post}}}{N_{\text{initial}}}\times 100$$

Here, *N*_*post*_ represents the postdigestion probiotic bacteria count, and *N*_*initial*_ represents the initial probiotic bacteria count.

### 2.9 Viability of Probiotic Bacteria Under Storage Conditions

The viability of free and encapsulated LR and LPC was assessed at three different storage temperatures (+24°C, +4°C, and −24°C) over a 28‐day period. Microbiological analysis were performed on Days 0, 7, 14, 21, and 28 to assess bacterial viability during storage. The impact of storage time on the viability of free (log cfu/mL) and encapsulated (log cfu/g) probiotic bacteria was determined using Equation (4).

## 3 Results and Discussion

### 3.1 Optimization of LR and LPC Encapsulation

**FIGURE 2:**
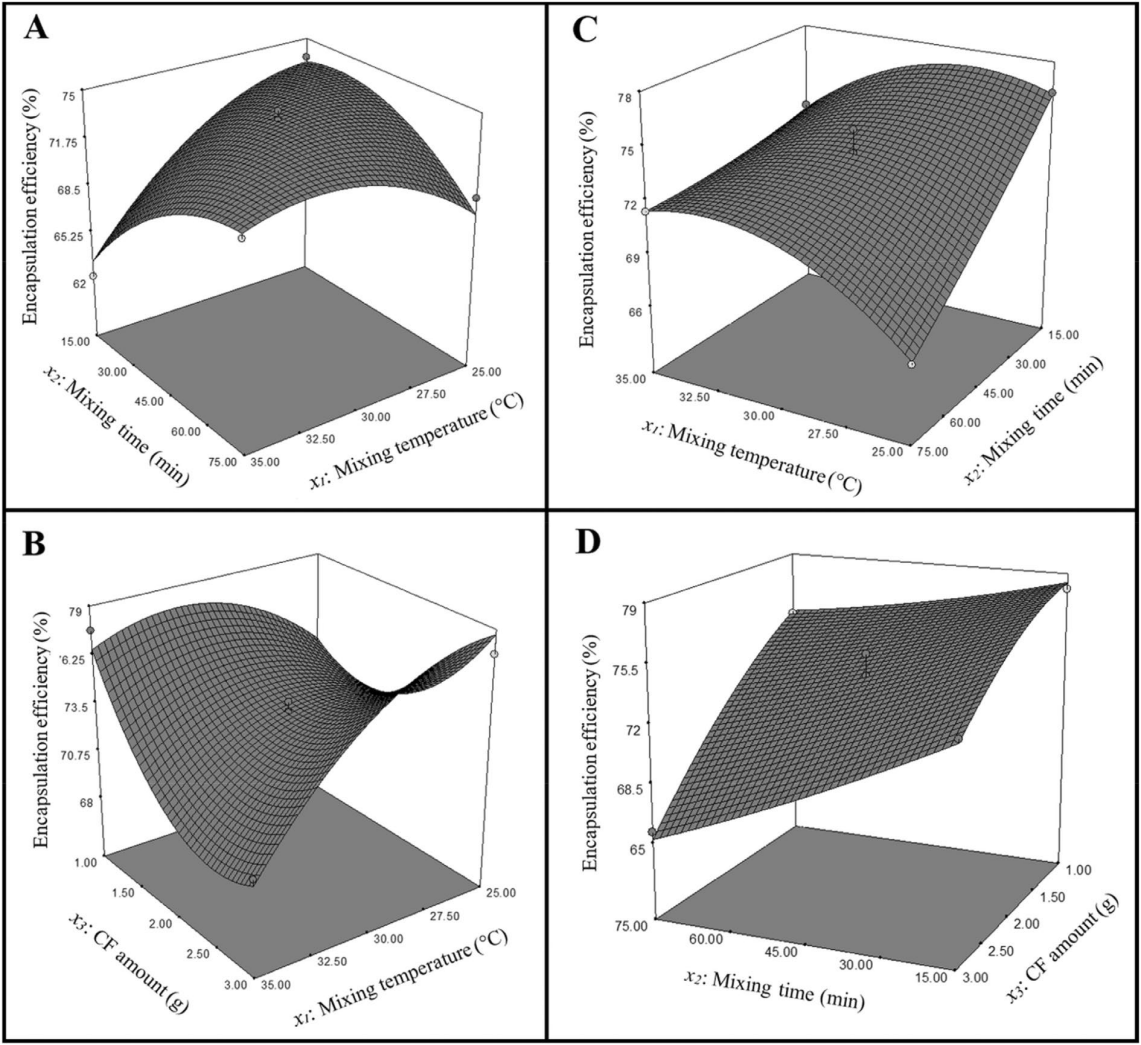
Three‐dimensional response surface plots for encapsulation efficiency: (A) Effect of mixing time and mixing temperature for *L. rhamnosus* , (B) Effect of carob flour amount and mixing temperature for *L. rhamnosus* , (C) Effect of mixing temperature and mixing time for *L. paracasei* , and (D) Effect of mixing time and carob flour amount for *L. paracasei*.

The encapsulation conditions were optimized to achieve the highest efficiency in encapsulating LR and LPC using CF. ANOVA results and second‐order polynomial equation for LR and LPC are in Table 3. For LR, the *p*‐value for the model was 0.0003, indicating a significant relationship between the response and the variables in the quadratic polynomial model (*p* < 0.0001). The model's *F*‐value was 20.21, which was deemed statistically significant at a 95% confidence level. The lack‐of‐fit *p*‐value for LR was 0.0763, suggesting that the lack‐of‐fit was not significant. For LPC, the model's *p*‐value was less than 0.0001, indicating a highly significant relationship between the response and the variables (*p* < 0.0001). The *F*‐value for the LPC model was 43.00, which was also considered significant at the 95% confidence level. The lack‐of‐fit *p*‐value for LPC was 0.7753, indicating that the model's lack‐of‐fit was not statistically significant. In the encapsulation of LR with CF, the significant factors affecting encapsulation efficiency (*p* < 0.05) were identified as *x*_*1*_, *x*_*2*_, *x*_*3*_, *x*_*1*_ *x*_*2*_, *x*_*1*_ *x*_*3*_, *x*_*1*_^2^, *x*_*2*_^2^, and *x*_*3*_^2^. Similarly, in the encapsulation of LPC with CF, the significant factors (*p* < 0.05) influencing encapsulation efficiency were *x*_*1*_, *x*_*2*_, *x*_*3*_, *x*_*1*_ *x*_*2*_, *x*_*2*_ *x*_*3*_, *x*_*1*_^2^, and *x*_*3*_^2^. The experimental encapsulation efficiency (%) obtained from the encapsulation process and the predicted encapsulation efficiency (%) determined via ANOVA analysis are presented in Table 4. Three‐dimensional response surface plots from ANOVA analysis for encapsulation efficiency of LR and LPC probiotic bacteria are presented in Figure 2. For LR, encapsulation efficiency increased as the mixing temperature approached 30°C when the mixing time was fixed at 75 min; however, it declined as the temperature rose to 35°C. At 35°C, a decrease in mixing time led to reduced efficiency. The highest efficiency was observed at a mixed temperature of 25°C with a mixing time of 15 min (Figure 2A). When the CF amount was kept constant at 3 g, increasing the temperature resulted in a decrease in efficiency. At 35°C, reducing the CF amount from 3 g to approximately 2.5 g decreased efficiency, but a further reduction in the CF amount led to an increase in efficiency. The highest efficiency was obtained with 3 g of CF amount at a mixing temperature of 25°C (Figure 2B). For LPC encapsulation efficiency, when the mixing time was fixed at 15 min, the efficiency increased as the temperature approached 27.5°C but decreased at higher temperatures. At 25°C, reducing the mixing time improved efficiency, with the maximum efficiency achieved by a mixing time of 15 min and a mixing temperature of 25°C (Figure 2C). When the CF amount was fixed at 3 g, efficiency decreased with increasing mixing time. Similarly, at a mixing time of 75 min, an increase in CF amount resulted in reduced efficiency. The highest efficiency was achieved with 1 g of CF amount and a mixing time of 15 min (Figure 2D). The optimum conditions and the predicted and experimental encapsulation efficiency under these conditions are summarized in Table 5. The experimental encapsulation efficiency for LPC was 74.98% ± 0.20%, whereas for LR, it was 79.51% ± 0.36%. The findings indicate that the BBD model provides a highly accurate and reliable estimation of maximum encapsulation efficiency for the encapsulation of LR and LPC using the emulsion method with CF.

**TABLE 3:** ANOVA for the response surface quadratic model and second‐order polynomial equation of *L. rhamnosus* and *L. rhamnosus* .

Source	LR (*R* ^*2*^ = 0.9629)					LPC (*R* ^*2*^ = 0.9822)				
	SS	df	MS	*F*	*p*	SS	df	MS	*F*	*p*
Model	201.21	9	22.36	20.21	0.0003	229.29	9	25.48	43.00	< 0.0001
Lack‐of‐fit	6.12	3	2.04	5.03	0.0763	0.91	3	0.30	0.38	0.7753
Pure error	1.62	4	0.41			3.23	4	0.81		
**Response**	**Second‐order polynomial equation**									
EE (%) LR	*y* = 73.67 − 1.65*x*_1_ + 0.94*x*_2_ − 0.59*x*_3_ + 3.44*x*_1_ *x*_2_ − 2.98*x*_1_ *x*_3_ − 2.35*x*_1_^2^ − 2.11*x*_2_^2^ + 3.36*x*_3_^2^									
EE (%) LPC	*y* = 73.66 + 0.40*x*_1_ − 2.75*x*_2_ − 3.87*x*_3_ − 2.01*x*_1_ *x*_2_ − 1.16*x*_2_ *x*_3_ − 2.17*x*_1_^2^ − 0.99*x*_3_^2^									

Abbreviations: df, degree of freedom; EE, encapsulation efficiency; LPC, *L. rhamnosus* ; LR, *L. rhamnosus* ; MS, mean square; SS, sum of squares; *x*_1_, mixing temperature (°C); *x*_2_, mixing duration (min); *x*_3_, carob flour amount (g).

**TABLE 4:** The predicted and experimental encapsulation efficiency of *L. rhamnosus* and *L. paracasei* (%).

Run order	Encapsulation efficiency (%)			
	LR		LPC	
	Experimental	Predicted	Experimental	Predicted
1	73.67	73.35	76.22	76.17
2	62.10	63.18	73.09	72.94
3	69.45	68.36	66.49	66.64
4	71.63	71.95	71.41	71.46
5	73.58	73.93	74.86	74.49
6	77.65	76.59	74.51	74.24
7	77.66	78.72	65.44	65.71
8	69.81	69.46	67.18	67.55
9	73.94	73.91	78.03	78.45
10	76.36	77.10	75.04	75.26
11	74.78	74.04	73.24	73.02
12	74.60	74.63	65.63	65.21
13	73.81	73.67	73.68	73.66
14	73.81	73.67	72.53	73.66
15	74.36	73.67	73.68	73.66
16	72.62	73.67	75.03	73.66
17	73.74	73.67	73.38	73.66

Abbreviations: LPC, *L. paracasei* ; LR, *L. rhamnosus* .

**TABLE 5:** Optimum conditions and predicted and experimental encapsulation efficiency for *L. rhamnosus* and *L. paracasei* .

Response	Optimum conditions			Maximum encapsulation efficiency	
	Mixing temperature (°C)	Mixing time (min)	Carob flour amount (g)	Predicted (%)	Experimental (%)
LR	26.97	33.24	2.93	78.32	79.51 ± 0.36
LPC	26.82	15.15	1.38	78.19	74.98 ± 0.20

Abbreviations: LPC, *L. paracasei* ; LR, *L. rhamnosus* .

Karkar et al. (2024) employed CCD‐RSM to investigate the impact of temperature, time, and coating material amount on probiotic encapsulation efficiency. For *L. casei* , the highest encapsulation efficiency was achieved at an extraction temperature of 36.5°C, an extraction time of 142.47 min, and a coating material concentration of 0.60%. For *L. acidophilus* , the maximum encapsulation efficiency was achieved at an extraction temperature of 36.5°C, an extraction time of 145.41 min, and a coating material concentration of 0.68%. As in this study, the strong agreement between model predictions and experimental results demonstrated the reliability of RSM‐based optimization approaches. The contrasting trends in temperature and coating material effects emphasized the importance of probiotic strain‐specific optimization. In another study by Rojas‐Espina et al. (2024), *L. plantarum* capsules were developed using different concentrations of CaCl_2_, chitosan, and inulin, optimized through BBD‐RSM. Under optimum conditions (1.84% CaCl_2_, 1.04% chitosan, and 1.4% inulin), the properties of capsules were effectively balanced with the impact of prebiotic supplementation, resulting in a high viability rate of 68.33%. The experimental and predicted results confirmed the model's validity, showing a strong correlation between bacterial viability values.

### 3.2 Physicochemical Composition of CF‐LR and CF‐LPC Capsules

The total sugar, fat, protein, dietary fiber, dry matter, moisture, acidity, and ash contents of CF, CF‐LR, and CF‐LPC capsules are presented in Table 6. The data obtained were analyzed using ANOVA via MINITAB 17.0 (Minitab Inc., State College, PA) statistical software, and pairwise comparisons for each physicochemical property were conducted at a significance level of *p* < 0.01. The total sugar content of CF was determined as 47.70% ± 0.67%, whereas it was not detectable in CF‐LR and CF‐LPC capsules. This result can be attributed to the fermentation of carbohydrates by probiotic bacteria as an energy source (Doğan 2011). Since CF has a very low‐fat content, the total fat amount remained below the detection limit, and this did not change with the presence of probiotic bacteria. The increase in protein content in CF‐probiotic bacteria capsules compared to CF (4.61% ± 0.25%) is attributed to the presence of probiotic biomass proteins, including cell wall proteins and enzymes (Chapot‐Chartier and Kulakauskas 2014). The higher protein content in CF‐LPC (17.56% ± 0.95%) capsules than CF‐LR (14.37% ± 0.78%) suggests that LPC may have denser biomass or a higher interaction capacity with CF. The dietary fiber content of CF was determined as 35.3% ± 1.35%, while it increased to 74.8% ± 2.86% and 74.9% ± 2.87% in CF‐LR and CF‐LPC capsules, respectively. This increase can be attributed to the conversion of some carbohydrates in CF into dietary fiber‐like structures during encapsulation. CF exhibited a high dry matter content of 97.66% ± 0.004%. The dry matter content of CF‐LR and CF‐LPC capsules was 95.44% ± 0.01% and 100.00% ± 0.01%, respectively. A slight decrease was observed in CF‐LR, while CF‐LPC reached 100%. This effect may be due to the direct influence of probiotics on moisture content or the lyophilization process. The acidity level was 1.02% ± 0.04% for CF, decreasing to 0.66% ± 0.03% and 0.60% ± 0.02% for CF‐LR and CF‐LPC capsules, respectively. This result may be attributed to the metabolic activity of probiotic bacteria, specifically their capacity to utilize carbohydrates during fermentation. The ash content of CF was relatively low (2.61% ± 0.20%). However, it increased to 3.39% ± 0.19% and 4.13% ± 0.09% in the CF‐LR and CF‐LPC capsules, respectively. The increase in ash content can be attributed to the accumulation of minerals because of probiotic metabolic activity (Khan et al. 2024). The higher ash content in CF‐LPC capsules indicates that LPC may have metabolized more minerals.

**TABLE 6:** Physicochemical properties of carob flour, carob flour–probiotic bacteria capsules.

Physicochemical property (%)	CF	CF‐LR	CF‐LPC
Total sugar	47.70 ± 0.67^B^	nd	nd
Total fat	nd	nd	nd
Total protein	4.61 ± 0.25^c,D^	14.37 ± 0.78^b,C^	17.56 ± 0.95^a,C^
Total dietary fiber	35.3 ± 1.35^b,C^	74.8 ± 2.86^a,B^	74.9 ± 2.87^a,B^
Dry matter	97.66 ± 0.004^b,A^	95.44 ± 0.01^c,A^	100.00 ± 0.01^a,A^
Moisture	2.34 ± 0.004^b,E,F^	4.56 ± 0.01^a,D^	nd
Acidity (acetic acid)	1.02 ± 0.04^a,F^	0.66 ± 0.03^b,E^	0.60 ± 0.02^b,E^
Ash	2.61 ± 0.20^c,E^	3.39 ± 0.19^b,D,E^	4.13 ± 0.09^a,D^

*Note:* Mean ± standard deviation (two replicate). a–c: superscript lowercase letters indicate statistically significant differences between samples for the same physicochemical property (*p* < 0.01). A–F: superscript uppercase letters indicate statistically significant differences between physicochemical properties within the same sample (*p* < 0.01).

Abbreviations: CF, carob flour; LPC, *L. paracasei*; LR, *L. rhamnosus*; nd, not detected.

A study by Konak et al. (2023) analyzed the chemical composition of carob flour. The results indicated that dry matter content was 95.44% ± 1.26%, crude protein was 7.28% ± 0.13%, fat content was 0.5% ± 0.01%, ash content was 3.11% ± 0.01%, and total dietary fiber was 47.14% ± 1.71%. According to a study by Ayad et al. (2023), the pulp of carob fruits collected from two different regions of Algeria was ground into powder. In the Djimla region, the carob pulp powder was found to contain a total titratable acidity of 1.16% ± 0.01%, a moisture content of 13.13% ± 0.003%, a dry matter content of 86.88% ± 0.005%, an ash content of 2.72% ± 0.002%, a protein content of 4.65% ± 0.09%, and a total sugar content of 26.8% ± 1.34%. In the El‐Milia region, the untreated carob pulp powder exhibited a total titratable acidity of 0.85% ± 0.007%, moisture content of 13.11% ± 0.005%, dry matter content of 86.89% ± 0.003%, ash content of 3.04% ± 0.001%, crude protein content of 4.79% ± 0.04%, and total sugar content of 27.7% ± 3.86%. In another study by Kılıç et al. (2022), the results demonstrated that yogurts produced with encapsulated lactic acid bacteria exhibited higher bacterial viability compared to those produced with free lactic acid bacteria on Days 1, 7, and 14 (11.21 ± 0.04 log cfu/g, 11.04 ± 0.15 log cfu/g, and 10.92 ± 0.03 log cfu/g, respectively). These yogurts also showed higher pH values (4.81 ± 0.01, 4.87 ± 0.03, and 4.93 ± 0.02, respectively) and higher dry matter content (17.82% ± 0.71%, 17.99% ± 0.59%, and 17.81% ± 0.19%, respectively). Additionally, yogurts containing encapsulated bacteria exhibited higher titratable acidity on Day 1 (0.65% ± 0.07%) but lower titratable acidity on Days 7 and 14 (0.62% ± 0.06% and 0.6% ± 0.05%, respectively).

### 3.3 Characterization of CF‐LR and CF‐LPC Capsules

#### 3.3.1 FTIR Analysis

**FIGURE 3:**
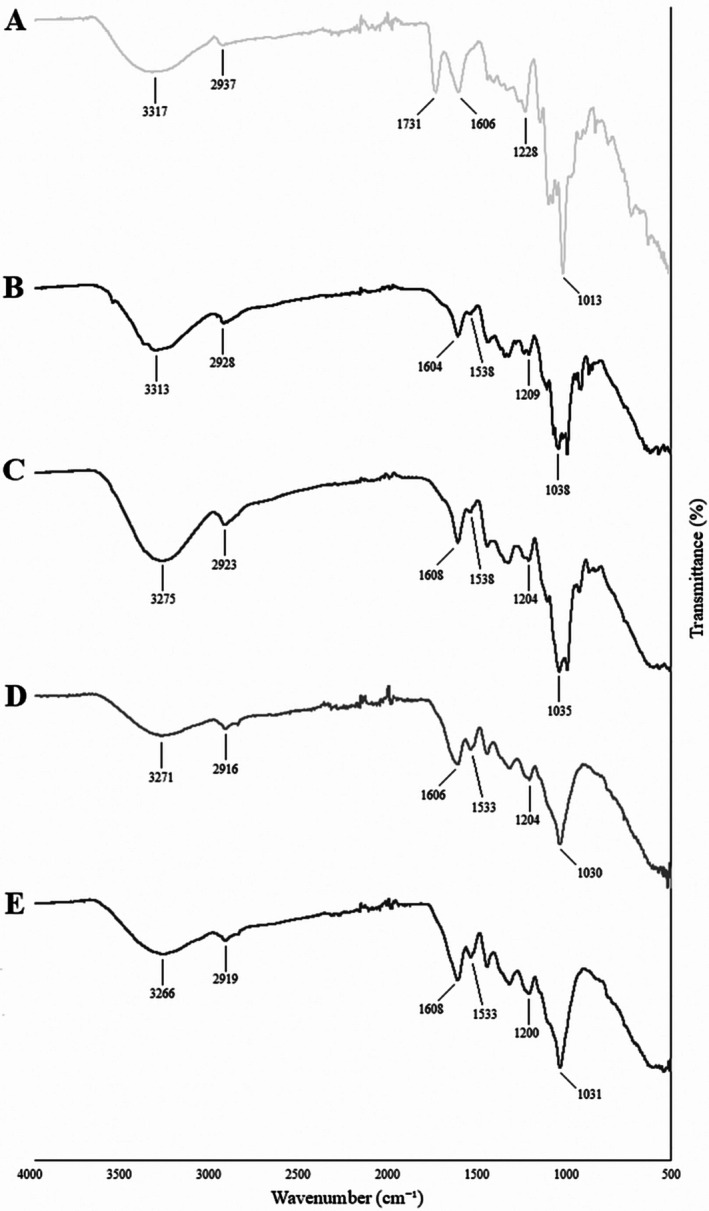
FTIR spectra: (A) Pectin, (B) sterilized carob flour, (C) carob flour, (D) carob flour‐ *L. paracasei* capsules, and (E) carob flour‐ *L. rhamnosus* capsules.

The FTIR spectra of pectin, CF, sterilized CF, CF‐LR, and CF‐LPC capsules used for structural characterization are presented in Figure 3. Upon analysis of the FTIR spectra, the absorption band within the 3400–3200 cm^−1^ range corresponds to the stretching vibration of O–H groups (Song et al. 2020). The 3000–2800 cm^−1^ region represents the C–H stretching vibration of methyl groups (Mahmud et al. 2022). The peaks associated with these spectral bands indicate significant structural changes following encapsulation (Figure 3D,E). A peak at 1731 cm^−1^ corresponds to the C=O vibration of the methyl ester group in pectin (Figure 3A). Peaks within the 1700–1500 cm^−1^ range represent amide I and II groups (Acordi Menezes et al. 2018; Demir et al. 2021; Karrar et al. 2021). Peaks between 1533 and 1731 cm^−1^ are associated with C=O and N–H vibrations in amides or protein structures. The broad absorption band in the 800–1200 cm^−1^ region corresponds to the carbohydrate fingerprint region, aligning with C–O and C–C stretching vibrations of polysaccharides. Following encapsulation, significant spectral changes were observed in the protein (1500–1600 cm^−1^) and carbohydrate (1000–1200 cm^−1^) regions, indicating that the probiotic bacteria successfully interacted with CF and integrated into its structure. The sterilization process did not induce substantial chemical changes in CF; only minimal spectral differences were observed (Figure 3B,C). However, the addition of probiotic bacteria resulted in more pronounced structural modifications, confirming their successful encapsulation.

#### 3.3.2 SEM Analysis

**FIGURE 4:**
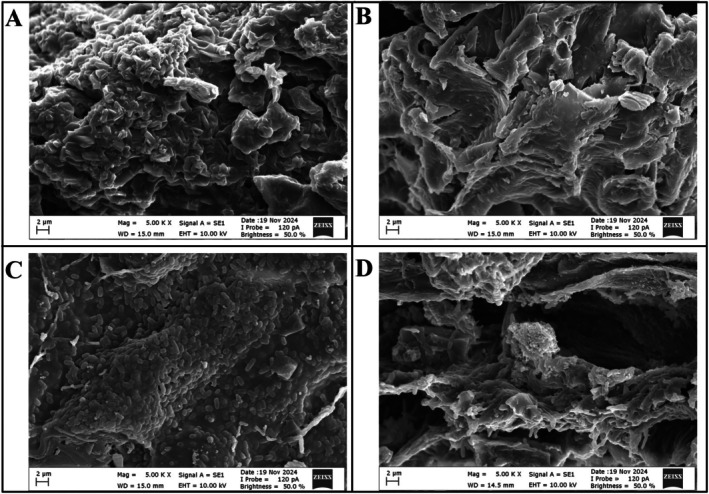
The SEM images: (A) Carob flour, (B) sterilized carob flour, (C) carob flour‐ *L. rhamnosus* capsules, and (D) carob flour‐ *L. paracasei* capsules.

The surface morphology of CF, sterilized CF, CF‐LR, and CF‐LPC capsules was examined using SEM (Figure 4). The SEM image of CF reveals an irregular surface morphology attributed to aggregations formed due to the structural composition (Figure 4A). Following sterilization, the high temperature and pressure weakened intermolecular interactions, leading to the disintegration of aggregations and the flattening of the CF surface (Figure 4B). The SEM images of CF‐LR and CF‐LPC capsules show protrusions formed by probiotic bacteria, indicating their successful embedding within the CF surface (Figure 4C,D). It is well known that freeze‐dried capsules exhibit a wrinkled, irregular, and collapsed surface morphology (Ivanovska et al. 2012). The porous surfaces observed after lyophilization are likely due to ice crystals or air bubbles trapped during freezing. The encapsulation process successfully facilitated the attachment of probiotic bacteria to the CF surface, altering its morphological structure and confirming the effective incorporation of probiotics into the matrix.

### 3.4 Viability of LR and LPC During In Vitro Gastrointestinal Digestion

**FIGURE 5:**
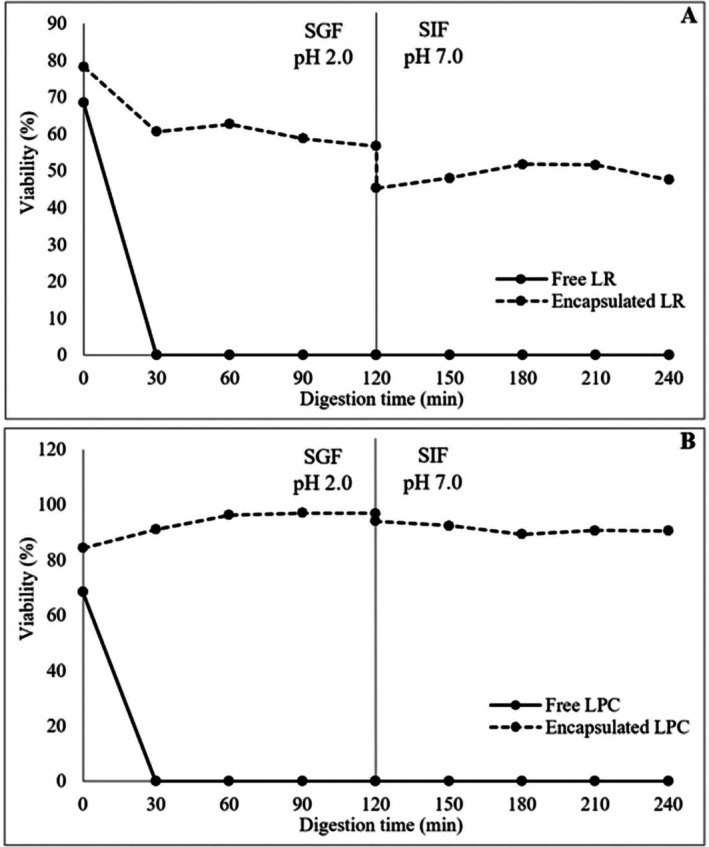
Effect of in vitro gastrointestinal digestion on the viability of *L. rhamnosus* (A) and *L. paracasei* (B).

The viability of free and encapsulated LR and LPC under in vitro gastrointestinal digestion conditions was investigated. The viable cell counts (log cfu/mL‐g) of free and encapsulated LR and LPC after in vitro gastrointestinal digestion are shown in Table 7. The effect of in vitro gastrointestinal digestion on the viability (%) of both free and encapsulated LR and LPC is shown in Figure 5. The viability of free LR and LPC decreased rapidly during gastric digestion, reaching undetectable levels after 60 min for LR and 30 min for LPC. However, probiotic capsules exhibited excellent resistance to the acidic environment and enzymatic activity of the SGF medium. At the end of 120 min of gastric digestion, the viability of LR capsules was maintained at 56.74%, while LPC capsules retained 96.93% viability. Free LR and LPC exhibited high viability rates during intestinal digestion, with viability levels of 106.13% and 85.35%, respectively. In encapsulated probiotic bacteria, viability was even higher, with LR capsules showing 111.48% viability and LPC capsules 108.36% viability. The neutral to mildly alkaline pH of the SIF medium, which simulates intestinal conditions, is known to be an optimal environment for the growth and viability of probiotic bacteria, particularly *Lactobacillus* and *Lacticaseibacillus* spp. (Stasiak‐Różańska et al. 2021). Moreover, the increase in live lactic acid bacteria observed under simulated intestinal conditions can be attributed to the prebiotic effects of pectin and CF used in the encapsulation matrix. These ingredients serve as fermentable substrates, enhancing bacterial viability and even promoting limited growth during the intestinal phase (Hamid et al. 2024). When subjected to sequential digestion in simulated gastric and intestinal environments, free LR and LPC exhibited a substantial loss of viability, reaching undetectable levels. However, viability was significantly preserved in encapsulated probiotics after the transition from the gastric to the intestinal environment. The viability rates after sequential digestion were 47.63% for LR capsules and 90.52% for LPC capsules, with LPC capsules exhibiting superior viability compared to LR capsules. This difference may be attributed to variations in strain‐specific resistance to gastrointestinal stress (Reale et al. 2015). Overall, the encapsulation process effectively protected probiotic bacteria throughout gastrointestinal digestion. The CF coating material successfully shielded probiotics from gastric acidity, facilitating their viability until they reached the intestinal environment. These findings highlight encapsulation technology as a crucial tool for enhancing probiotic resistance to gastrointestinal digestion. Additionally, the combination of probiotic encapsulation with prebiotic wall matrix materials may lead to the development of symbiotic capsules, thereby improving probiotic efficacy and contributing to advancements in the functional food industry (Ta et al. 2021; Soto et al. 2023).

**TABLE 7:** Viability of *L. rhamnosus* and *L. paracasei* after in vitro gastrointestinal digestion.

Medium	Digestion time (min)	Free^a^ LR	Encapsulated^b^ LR	Free^a^ LPC	Encapsulated^b^ LPC
	İnitial	7.01 ± 0.01	5.49 ± 0.01	8.67 ± 0.01	5.38 ± 0.01
SGF	0	4.71 ± 0.01	4.30 ± 0.14	5.94 ± 0.19	4.54 ± 0.06
	30	1.50 ± 0.14	3.33 ± 0.07	nd	4.90 ± 0.11
	60	nd	3.45 ± 0.05	nd	5.18 ± 0.10
	90	nd	3.23 ± 0.11	nd	5.22 ± 0.01
	120	nd	3.12 ± 0.12	nd	5.22 ± 0.02
SIF	0	7.11 ± 0.03	5.21 ± 0.13	6.99 ± 0.17	5.07 ± 0.13
	30	7.34 ± 0.08	5.84 ± 0.01	7.20 ± 0.15	5.52 ± 0.13
	60	7.24 ± 0.01	5.90 ± 0.05	6.94 ± 0.06	5.77 ± 0.01
	90	7.44 ± 0.08	6.09 ± 0.14	7.24 ± 0.15	5.68 ± 0.01
	120	7.44 ± 0.10	6.15 ± 0.04	7.40 ± 0.13	5.83 ± 0.08
SGF + SIF	0	4.71 ± 0.01	4.30 ± 0.14	5.94 ± 0.19	4.54 ± 0.06
	30	1.50 ± 0.14	3.33 ± 0.18	nd	4.90 ± 0.11
	60	nd	3.45 ± 0.05	nd	5.18 ± 0.10
	90	nd	3.23 ± 0.11	nd	5.22 ± 0.01
	120	nd	3.12 ± 0.16	nd	5.22 ± 0.02
	120	nd	2.49 ± 0.16	nd	5.06 ± 0.16
	150	nd	2.64 ± 0.08	nd	4.98 ± 0.09
	180	nd	2.85 ± 0.08	nd	4.81 ± 0.12
	210	nd	2.84 ± 0.23	nd	4.88 ± 0.14
	240	nd	2.62 ± 0.02	nd	4.87 ± 0.13

*Note:* Mean ± standard deviation (two replicate).

Abbreviations: LPC, *L. paracasei* ; LR, *L. rhamnosus* ; nd, not detected; SGF, simulated gastric fluid; SIF, simulated intestinal fluid.

^a^ Log cfu/mL.

^b^ Log cfu/g.

In a study conducted by Zubair et al. (2023), the viability of both free and encapsulated *L. rhamnosus* was evaluated under simulated gastrointestinal conditions. The study recorded a reduction in probiotic viability of 7.84 log cfu/mL for free bacteria, 1.78 log cfu/mL for bacteria encapsulated with taro starch, and 2.81 log cfu/mL for those encapsulated with alginate. After 120 min in simulated gastric fluid (SGF) and simulated intestinal fluid (SIF), the highest viability was observed in probiotics encapsulated with taro starch, showing viability levels of 8.21 ± 0.02 log cfu/mL in SGF and 7.61 ± 0.01 log cfu/mL in SIF. In another study by Xiao et al. (2020), the viability of *L. bulgaricus* and *L. paracasei* was assessed under similar gastrointestinal conditions after encapsulation using a layer‐by‐layer approach with milk protein isolate and combinations of milk protein isolate with xanthan gum. The viability reduction for free probiotics in SGF was 5.02 log cfu/mL. In contrast, probiotics encapsulated with milk protein isolate exhibited a 2.11 log cfu/g reduction, while those encapsulated with the milk protein isolate‐xanthan gum combination showed a 1.34 log cfu/g reduction. Free probiotic bacteria experienced a 5.94 log cfu/mL loss in the bile solution. In comparison, those encapsulated with milk protein isolate showed a 3.47 log cfu/g reduction, and those with milk protein isolate and xanthan gum showed a 2.27 log cfu/g reduction. SIF was less detrimental to probiotic viability, with both free and encapsulated bacteria maintaining viability above 7 log cfu/mL‐g. A study by de Matos‐Jr et al. (2019) evaluated the tolerance of two probiotic strains immobilized in gelatin and arabic gum‐coated solid lipid microparticles under simulated gastrointestinal conditions. Free *L. paracasei* BGP1 and *L. rhamnosus* 64 exhibited significant sensitivity to SGF, with a viability reduction of 1.31 log cfu/mL for *L. rhamnosus* 64 and 3.37 log cfu/mL for *L. paracasei* BGP1. However, exposure to SIF had a lesser impact, with viability reductions of 1.64 log cfu/mL for *L. rhamnosus* 64 and 1.71 log cfu/mL for *L. paracasei* BGP1 after 180 min of digestion. In SGF, the viability reduction for encapsulated *L. paracasei* BGP1 was 0.66 log cfu/g, while for encapsulated *L. rhamnosus* 64, it was 1.13 log cfu/g. In SIF, the viability loss was 0.75 log cfu/g for encapsulated *L. paracasei* BGP1 and 0.63 log cfu/g for encapsulated *L. rhamnosus* 64.

### 3.5 Viability of LR and LPC Under Storage Conditions

**FIGURE 6:**
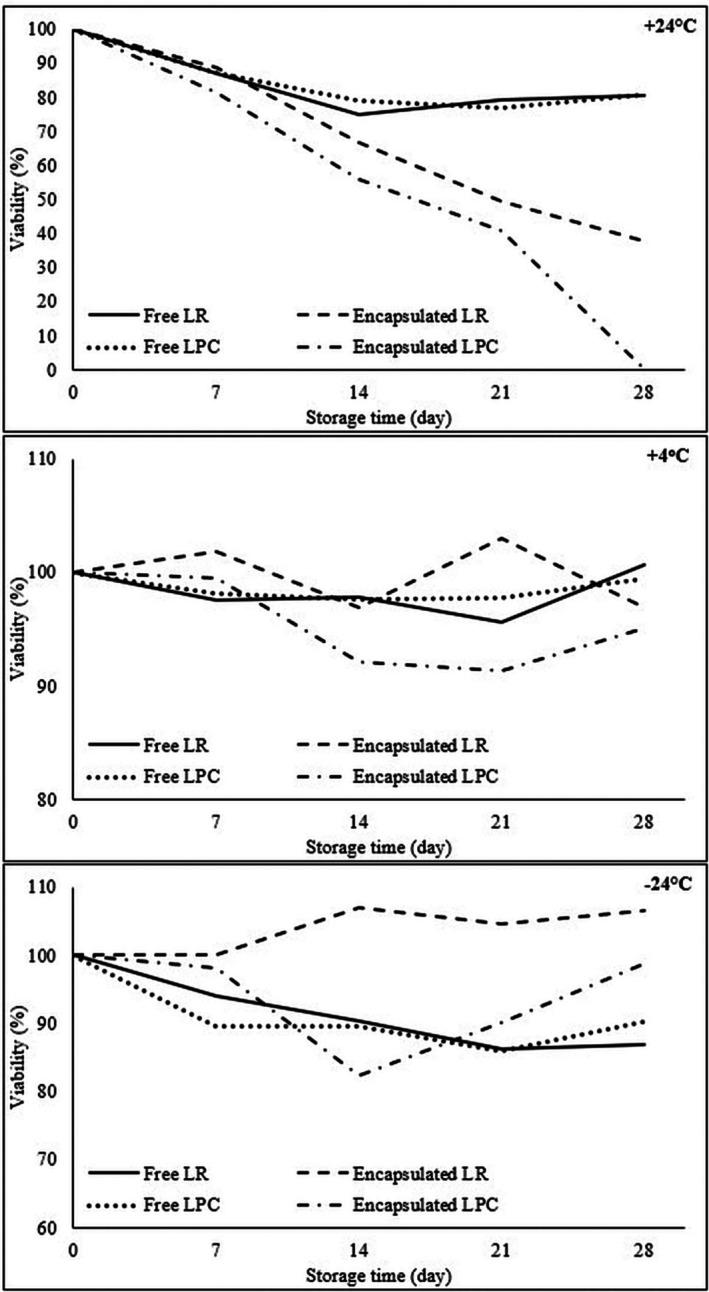
The viability of *L. rhamnosus* and *L. paracasei* during storage at different temperatures.

The viable cell counts (log cfu/mL‐g) of free and encapsulated LR and LPC after storage conditions are given in Table 8. The effects of storage time and temperature on the viability (%) of free and encapsulated LR and LPC are graphically illustrated in Figure 6, respectively. At +24°C, the viability of free LR and LPC gradually decreased throughout storage, reaching 80.40% ± 0.24% and 80.67% ± 0.32%, respectively, by Day 28. However, in encapsulated probiotics, the temperature impact was more pronounced, leading to a faster decline in viability. By the end of Day 28, the viability of CF‐LR capsules dropped to 37.83%, whereas CF‐LPC viability was undetectable. The encapsulated probiotics exhibited lower resistance to +24°C compared to free probiotics, likely due to the degradation of the natural coating material at room temperature, which diminished its protective effect on bacterial cells (Higl et al. 2007; Heidebach et al. 2010). At +4°C, the viability of free LR and LPC remained above 95% throughout the 28‐day storage period. Similarly, the viability of encapsulated LR and LPC on Day 28 was recorded as 96.88% ± 2.56% and 95.05% ± 1.56%, respectively. At −24°C, a lower reduction in viability was observed in free LR and LPC, with viability rates of 86.99% ± 1.62% for LR and 90.30% ± 0.73% for LPC by Day 28. Encapsulated probiotic bacteria exhibited the highest viability rates at −24°C, with viability levels of 106.50% ± 0.12% for CF‐LR and 98.74% ± 1.22% for CF‐LPC on Day 28. In contrast, encapsulation effectively preserved the stability of probiotics at +4°C and −24°C, as indicated by higher viable cell counts throughout storage. LR demonstrated superior resistance to storage conditions compared to LPC, with −24°C storage enhancing the stability of CF‐probiotic bacteria capsules, resulting in increased viable cell counts.

**TABLE 8:** Viability of *L. rhamnosus* and *L. paracasei* after storage conditions.

Temperature (°C)	Storage time (day)	Free^a^ LR	Encapsulated^b^ LR	Free^a^ LPC	Encapsulated^b^ LPC
+24	0	8.73 ± 0.04	6.08 ± 0.24	8.78 ± 0.01	6.37 ± 0.04
	7	7.58 ± 0.08	5.41 ± 0.14	7.65 ± 0.08	5.17 ± 0.18
	14	6.53 ± 0.01	4.07 ± 0.11	6.92 ± 0.08	3.57 ± 0.12
	21	6.92 ± 0.11	3.00 ± 0.14	6.76 ± 0.08	2.60 ± 0.01
	28	7.02 ± 0.02	2.30 ± 0.01	7.08 ± 0.03	nd
+4	0	8.73 ± 0.04	6.08 ± 0.24	8.78 ± 0.01	6.37 ± 0.04
	7	8.51 ± 0.01	6.20 ± 0.18	8.62 ± 0.06	6.33 ± 0.18
	14	8.54 ± 0.06	5.90 ± 0.16	8.57 ± 0.03	5.86 ± 0.23
	21	8.34 ± 0.04	6.27 ± 0.18	8.58 ± 0.08	5.81 ± 0.16
	28	8.78 ± 0.03	5.89 ± 0.58	8.73 ± 0.01	6.05 ± 0.10
−24	0	8.73 ± 0.04	6.08 ± 0.24	8.78 ± 0.01	6.37 ± 0.04
	7	8.20 ± 0.11	6.08 ± 0.07	7.87 ± 0.04	6.24 ± 0.06
	14	7.88 ± 0.04	6.51 ± 0.02	7.86 ± 0.09	5.24 ± 0.08
	21	7.52 ± 0.14	6.37 ± 0.01	7.55 ± 0.19	5.74 ± 0.11
	28	7.59 ± 0.14	6.48 ± 0.01	7.93 ± 0.06	6.29 ± 0.08

*Note:* Mean ± standard deviation (two replicate).

Abbreviations: LPC, *L. paracasei* ; LR, *L. rhamnosus* ; nd, not detected.

^a^ Log cfu/mL.

^b^ Log cfu/g.

A study by Nuaman et al. (2024) investigated the viability of *L. rhamnosus* GG encapsulated in sodium alginate, arabinoxylan, and a combination of both materials under storage conditions. On Day 1, the viability of *L. rhamnosus* capsules was measured at 8.9 log cfu/g. After 28 days of storage at 5°C, a loss of approximately 2 log cfu/g in viability was observed. The incorporation of arabinoxylan enhanced the structure of the polymer network through the formation of covalent cross‐links, which improved bacterial viability. Consequently, the capsules of *L. rhamnosus* that were encapsulated with sodium alginate‐arabinoxylan exhibited the highest viability rate, measuring 6.6 log cfu/g by Day 28.

In another study by Fredua‐Agyeman (2024), *L. acidophilus* LA‐5 was freeze‐dried using ringer solution, glycerol, skim milk, and trehalose and stored at 4°C and 25°C. At 4°C, the cells preserved with ringer solution lost viability immediately, while those preserved with glycerol lost viability within 7 days. However, cells maintained with skim milk or trehalose remained viable for up to 2 months, with skim milk showing higher viability over a 6‐month storage period. At 25°C, the ringer solution also resulted in immediate loss of viability, and glycerol‐preserved cells lost viability within 3 days. Although both skim milk and trehalose preserved viability for the first 14 days, only trehalose provided better protection for up to 6 months.

Additionally, a study examining the storage of wet and dry capsules of *B. lactis* Bb‐12, made with gelatin and gum arabic, evaluated their storage at 25°C, 7°C, and −18°C. At 25°C, both wet and dry capsules retained their viability for 90 days, showing viable counts of 6.60 log cfu/g and 9.45 log cfu/g, respectively. However, a significant decline was noted after 120 days. At 7°C, both capsule forms maintained viability above 6 log cfu/g throughout the 120‐day period. By the end of the storage duration, the viability of wet capsules decreased by 5.64 log cfu/g, while dry capsules exhibited a reduction of 6.09 log cfu/g. Storage at −18°C ensured the highest viability compared to other temperature conditions, with both wet and dry capsules maintaining viable counts above 6 log cfu/g after 120 days (da Silva et al. 2018).

## 4 Conclusion

This study demonstrated the effectiveness of CF as a natural encapsulation material for enhancing the stability and viability of probiotic bacteria during in vitro gastrointestinal digestion and storage conditions. The optimization of encapsulation conditions using the Box–Behnken Design ensured maximum encapsulation efficiency, with parameters such as mixing temperature, mixing time, and CF amount significantly influencing the viability of the probiotics. The encapsulation efficiencies of *L. rhamnosus* and *L. paracasei* capsules were determined as 79.51% ± 0.36% and 74.98% ± 0.20%, respectively. The physicochemical analysis confirmed that the encapsulation process altered the contents of protein, sugar, and dietary fiber. FTIR analysis revealed that the encapsulation process altered the chemical structure of CF in terms of specific functional groups while preserving its fundamental structural components. Additionally, the encapsulation process successfully facilitated the attachment of probiotic bacteria to the CF surface, resulting in morphological changes. Encapsulation effectively protected probiotics from gastric environments, as evidenced by higher viability rates in simulated gastrointestinal fluids than in free bacteria. Moreover, storage studies revealed that encapsulated probiotics maintained superior viability, particularly at refrigerated and frozen conditions, whereas higher temperatures led to a decline in bacterial viability. These findings emphasize the potential of CF as a functional encapsulation material for probiotic applications, supporting its use in the development of probiotic formulations for functional food and nutraceutical industries. Future studies should investigate additional prebiotic carriers and alternative encapsulation methods to enhance probiotic viability and efficacy further.

## Author Contributions

**Elif Tülek:** data curation (equal), formal analysis (equal), investigation (equal), validation (equal), writing – original draft (equal). **Büşra Karkar:** conceptualization (equal), data curation (equal), formal analysis (equal), investigation (equal), methodology (equal), supervision (equal), validation (equal), writing – original draft (equal), writing – review and editing (equal). **Saliha Şahin:** conceptualization (equal), data curation (equal), formal analysis (equal), funding acquisition (equal), investigation (equal), methodology (equal), resources (equal), software (equal), supervision (equal), validation (equal), visualization (equal), writing – review and editing (equal). **Lütfiye Yılmaz‐Ersan:** data curation (equal), formal analysis (equal), investigation (equal), methodology (equal), writing – review and editing (equal). **Ekin Sucu:** data curation (equal), formal analysis (equal), methodology (equal), writing – original draft (equal).

## Conflicts of Interest

The authors declare no conflicts of interest.

## Data Availability

The data that support the findings of this study are available on reasonable request from the corresponding author.
